# A Compend of Equine Anatomy and Physiology

**Published:** 1891-04

**Authors:** 


A Compend of Equine Anatomy and Physiology. By William B. Ballou, M. D., Professor of Equine Anatomy, and formerly lecturer on Physiology, New York College of Veterinary Surgeons. Instructor in Genito-Urinary Surgery, New York Polyclinic; Surgeon to Bellevue Dispensary; late House Surgeon Bellevue Hospital, New York. With twenty-nine graphic illustrations, selected from Chauveau's Comparative Anatomy. Quiz Compends No. 12. Philadelphia: P. Blakiston, Son & Co.

There is a deal of information upon the anatomy of the horse contained in this small book. There is, however, one important and rather serious omission, viz., that of dimension. In many instances only weight and capacity are mentioned, and often nothing at all. In many readers' minds the first thought is how does this or that organ compare with that of man in point of size, and this otherwise capital little book does not give the information. It is a handy little work to have within reach, and doubtless will be thoroughly appreciated. J. P.

